# Frazzled can act through distinct molecular pathways in epithelial cells to regulate motility, apical constriction, and localisation of E-Cadherin

**DOI:** 10.1371/journal.pone.0194003

**Published:** 2018-03-08

**Authors:** Sofia Golenkina, Vishal Chaturvedi, Robert Saint, Michael J. Murray

**Affiliations:** School of BioSciences, University of Melbourne, Melbourne, Victoria, Australia; National Institutes of Health, UNITED STATES

## Abstract

Netrin receptors of the DCC/NEO/UNC-40/Frazzled family have well established roles in cell migration and axon guidance but can also regulate epithelial features such as adhesion, polarity and adherens junction (AJ) stability. Previously, we have shown that overexpression of *Drosophila* Frazzled (Fra) in the peripodial epithelium (PE) inhibits wing disc eversion and also generates cellular protrusions typical of motile cells. Here, we tested whether the molecular pathways by which Fra inhibits eversion are distinct from those driving motility. We show that in disc proper (DP) epithelial cells Fra, in addition to inducing F-Actin rich protrusions, can affect localization of AJ components and columnar cell shape. We then show that these phenotypes have different requirements for the three conserved Fra cytoplasmic P-motifs and for downstream genes. The formation of protrusions required the P3 motif of Fra, as well as integrins (*mys* and *mew*), the Rac pathway (*Rac1*, *wave* and, *arpc3*) and myosin regulatory light chain (*Sqh*). In contrast, apico-basal cell shape change, which was accompanied by increased myosin phosphorylation, was critically dependent upon the P1 motif and was promoted by *RhoGef2* but inhibited by *Rac1*. Fra also caused a loss of AJ proteins (DE-Cad and Arm) from basolateral regions of epithelial cells. This phenotype required all 3 P-motifs, and was dependent upon the polarity factor *par6*. *par6* was not required for protrusions or cell shape change, but was required to block eversion suggesting that control of AJ components may underlie the ability of Fra to promote epithelial stability. The results imply that multiple molecular pathways act downstream of Fra in epithelial cells.

## Introduction

In recent years, it has become clear that cell surface receptors that mediate motility and guidance of migrating cells and axons can also play a role in epithelial morphogenesis events [1]. A case in point are the Netrin receptors of the Deleted in Colorectal Carcinoma (DCC) / Neogenin / UNC-40 / Frazzled family [2–7]. Netrins are a highly conserved family of secreted proteins, that can either attract or repel growing axons and migrating cells depending on which receptors are involved. DCC-family receptors normally mediate attraction but can also cause repulsion when paired with UNC5-family receptors [3,8,9]. During chemoattraction DCC-family receptors act through Src family kinases and Rho GTPases to promote cell protrusions by regulating the F-Actin cytoskeleton (reviewed in [10]). DCC can also act as a dependence receptor, promoting apoptosis in the absence of its ligand [11]. Furthermore, like Notch receptors, DCC-family receptors can undergo ectodomain shedding and gamma-secretase cleavage, allowing the intracellular domain to translocate to the nucleus where it can activate transcription [12–14].

Although DCC-family receptors are best known for their roles in neurons they can also regulate epithelial plasticity events (reviewed in [10,15,16]). For example, Netrin-1 and Neogenin appear to play an adhesive role in maintaining the structure of the proliferative and invasive terminal end buds during mammary gland development [17]. In *Drosophila*, we have found that Netrins and Frazzled regulate two epithelial events: the dissociation of the peripodial epithelium of wing discs during eversion [18] and the formation of the midgut epithelium during embryonic development [19]. In both cases, Fra promotes both motile and epithelial characteristics. In peripodial cells Fra can induce extensive protrusions, but can also inhibit the dissociation (i.e. partial EMT) of the peripodial epithelium (unpublished data) and subsequent eversion of the wing [18]. Similarly, in the midgut, Fra is required for midgut cells to migrate, and extend motile protrusions, but is also needed for apico-basal polarization and formation of an epithelium [19].

Thus, the question arises: when Fra is promoting epithelial phenotypes, is it acting through the same molecular pathways that mediate migration in motile cells. This is a distinct possibility given recent findings in a human epithelial cell line. In human intestinal colorectal cancer cells Neogenin promotes AJ stability by recruiting and activating components of the WAVE F-Actin polymerization pathway through Rac1 [20], a signaling pathway that plays a well-known role in axonal extension. Arguing against this, we have previously found that the two phenotypes associated with *fra* overexpression in the peripodial epithelium have different requirements in terms of *fra* expression levels. For protrusions, the more strongly *fra* is expressed the more prevalent the protrusions. For blocking eversion, however, intermediate levels of *fra* were most effective [18]. These observations suggested that there may be distinct molecular pathways driving each phenotype. In this paper, we provide more direct evidence for multiple pathways by firstly establishing a range of phenotypes in epithelial cells associated with Fra overexpression, and then showing that these are separable in terms of their requirement for particular regions of the Fra protein, or for different downstream genes.

To assess the importance of different regions of Fra we focussed on the three highly conserved, cytoplasmic P-motifs that are a characteristic of DCC-family receptors: P1, P2 and P3 [4,21]. Of the three, the P3 motif appears most important for chemoattraction. DCC gain-of-function analysis in *Xenopus laevis* spinal cord neurons indicated that the P3 motif is required for growth cone attraction to Netrin-1 [22] while in *Drosophila*, midline crossing of commissural neurons depends only on the P3 motif [23]. In vertebrates, the P3 domain is required for binding of FAK, which becomes activated in response to Netrin-1 [24–26]. It is also necessary for self-association and interaction with the Robo receptor [27]. In *Drosophila*, however, multimerization of Fra does not appear to depend on the P3 motif [23] and it is unknown whether FAK binding occurs. The P3 motif also interacts with the FERM-domain of Myosin-X which serves to recruit DCC to the tips of neurites growth cones, where the receptor regulates Myosin-X-mediated formation and elongation of basal filopodia [28]. The P3 motif of *Drosophila* Frazzled is also required for a synergistic interaction with Rho1 leading to activation of another member of the myosin family, non-muscle myosin II [29]. Finally the P3-motif is responsible for the transcriptional activity of the Fra Intracellular motif [14].

The roles of the P1 and P2 motifs are more enigmatic. In *C*. *elegans* an UNC-40 gain-of-function study showed that the P1 and P2 motifs, but not the P3, was required for excessive outgrowth, misguidance, branching, and deformed cell bodies of mechanosensory and motor neurons [30]. Further analysis indicated that both the P1 and P2 motifs promote actin rearrangements but the P1 acts through the recruitment of Unc-34/Enabled, whereas the P2 acts via the activation of Ced-10/Rac1 and Unc-115 (an actin-binding protein) [30]. *Drosophila* Ena and Trio, a Rac/Rho GEF, act downstream of Frazzled during attractive midline axon guidance, but specific requirements of the P motifs have not been investigated [31]. Interestingly, expression of a *fra* transgene lacking the P1 motif can cause axonal projection errors leading to speculation that the P1 motif may activate other molecular pathways that have an inhibitory effect on normal Fra activity [32]. The P1 motif also interacts with the eIFs and small ribosomal subunits in axons and dendrites [33] to regulate translation, and Unc5 to regulate growth cone repulsion [9]. The P2 motif also harbors a WIRS motif that can recruit the WAVE regulatory complex [20]. The P2 and P3 motifs have also been implicated in microtubule dynamics during axon pathfinding: both motifs of DCC were reported to bind directly to TUBB3 [34], a neuronal β-tubulin isotype III, which is known to be an essential factor mediating axon guidance during the CNS development [35].

Here we show that the ability of Fra to create motile protrusions in epithelial cells is most dependent upon its P3 domain and acts through the Rac1 Rho GTPase. In contrast, induction of apical constriction, requires the P1 domain and activates Rho1 contractile pathways. Finally, disruption of normal DE-Cad localisation and eversion failure requires the entire Fra protein and depends upon *par6*.

## Results

### Fra-dependent eversion failure requires full-length Fra and *par6*

**Fig 1:**
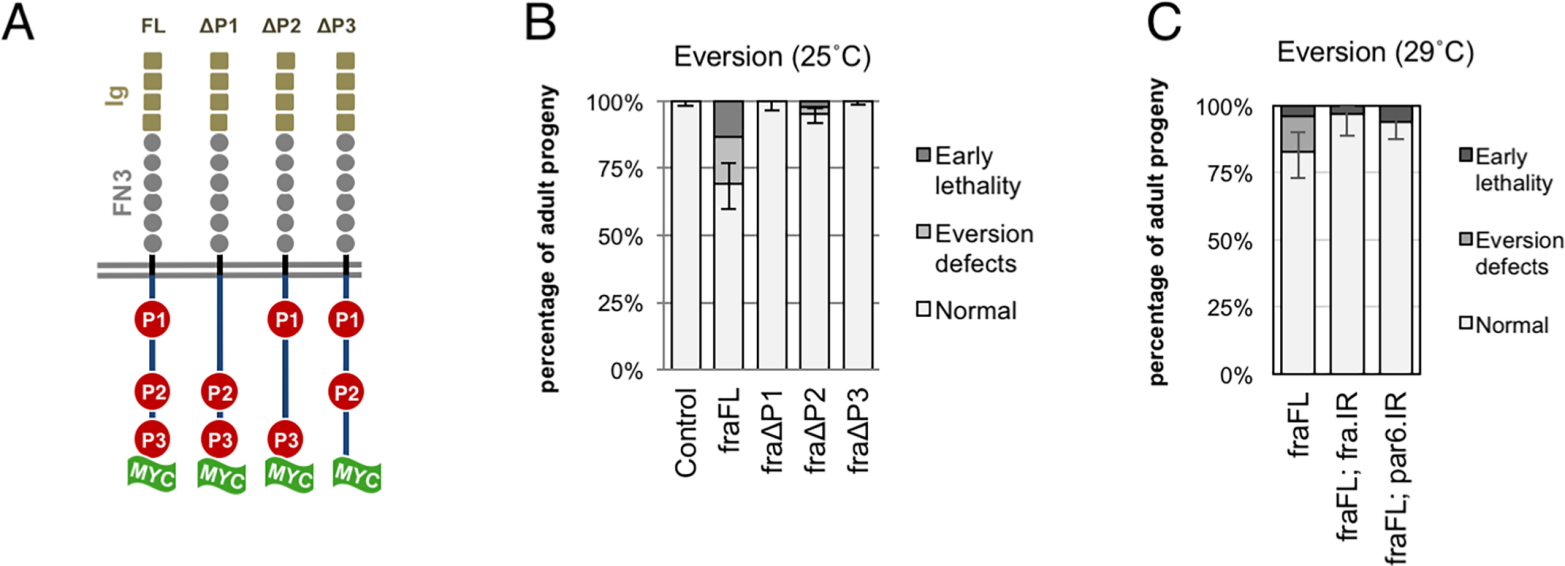
Adult eversion failure requires expression of full-length Fra and *par6*. (A) Schematic of myc-tagged *fra* deletion transgenes [23]. (B, C) Adult eversion proportions when the indicated transgenes are expressed using the *Ubx-GAL4* peripodial driver at 25°C (B) or *Ubx-GAL4*, *gal80ts* at 29°C (C). (B) Expression of *fraFL* disrupts eversion in ~30% of discs. Only *fra∆P2-myc* is also able to block eversion but is much less efficient than full-length *fra*. (C) *Ubx>fraFL-myc and Ubx>netA*.IR defects can be largely rescued by co-expression of either *par6*.*IR* or *fra*.*IR*. Error bars show 95% CI for the proportion of discs with normal eversion (by Wilson Score method).

We began our analysis of *fra* overexpression phenotypes by determining which P-motifs were required for eversion failure. To do this we compared the effects of expressing a full-length *UAS-fraFL-Myc* transgene with forms lacking either the P1, P2 or P3 motifs (*UAS-fra∆P1-Myc*, *UAS-fra∆P2-Myc*, *UAS-fra∆P3-Myc*) [23] in PE cells (Fig 1A). *Ubx-GAL4* expression of full-length *fraFL-myc* at 25°C produced ~30% progeny with eversion defects or early pupal lethality: 8.3% of progeny had uneverted wings, 9.2% displayed thoracic clefts, while 13.3% were lethal (n = 120; Fig 1B, Table 1). Of the three deletion transgenes only *fra∆P2-myc* produced eversion phenotypes, though the penetrance was much reduced to 5% (n = 396). Of the adults expressing *fra∆P2-myc*, 0.8% of flies had a missing wing, 1.6% displayed thoracic defects, and 2.5% were lethal at pupal stages. In contrast, expression of *fra∆P1-myc and fra∆P3-myc* had little to no effect on eversion (n = 110, n = 278 respectively).

**Table 1:** Adult wing disc eversion defects.

Genotype	No defects (%)	Eversion defects (%)	Early lethality (%)	Total (n)	p-value
***+;+;Ubx-GAL4/+***	100	0	0	214	-
***fraFL-MYC/+;+;Ubx-GAL4/+***	69.2	17.5	13.3	120	< 0.0001
***fra∆P1-MYC/+;+;Ubx-GAL4/+***	100	0	0	110	1
***fra∆P2-MYC/+;+;Ubx-GAL4/+***	95.1	2.4	2.5	396	< 0.0001
***fra∆P3-MYC/+;+;Ubx-GAL4/+***	99.7	0.3	0	278	1

All crosses performed at 25°C. p-values show Fisher's exact test of normal vs disrupted proportions with respect to *+;+;Ubx-GAL4/+* control genotype.

Next, we sought to identify other genes acting downstream of Fra during eversion. Using a *Ubx-GAL4*, *gal80*^*ts*^ driver we screened 49 genes for those that, when knocked down in late larval stages, could suppress *fra* over-expression adult eversion defects. These crosses were performed at 29°C which is not optimal for Fra-induced eversion defects [18], but was required for maximal RNAi effect. These genes were chosen to include genes implicated in netrin signaling, adhesion and cytoskeletal regulation, as well as epithelial polarity (S1 Table). Unfortunately, for most genes, RNAi knockdown generated extensive larval/pupal lethality, or strongly enhanced the Fra eversion failure rate. Only knockdown of *par6*, and *fra* itself, could repress eversion defects. The proportion of normal flies increased from 83% (*Ubx>fraFL-myc*) to 94% (*Ubx>fraFL-myc*, *par6-IR*) (p = 0.026; Fig 1C, Table 2). This degree of rescue was comparable to the effects of *fra*.*IR* itself where the proportion of normal flies increased to 97% (*Ubx>fra*, *par6-IR*) (p = 0.012; Fig 1C, Table 2).

**Table 2:** *par6* RNAi rescues eversion defects.

Genotype	No defects (%)	Eversion defects (%)	Early lethality (%)	Total (n)	p-value
*UAS-fraFL-myc/+;+;Ubx-GAL4*, *GAL80ts* */ +*	83	13	4	75	-
*UAS-fraFL-myc/+;+; Ubx-GAL4*, *GAL80ts /* *UAS-fra*.*IR*	97	0	3	60	0.012
*UAS-fraFL-myc/+;+; Ubx-GAL4*, *GAL80ts /* *UAS-par6*.*IR*	94	0	6	100	0.026

All crosses were performed at 29°C. p-values represent Fisher’s exact test values of normal progeny *vs* abnormal (i.e eversion defects or early pupal lethality) compared to control.

Thus, efficient disruption of eversion requires all three P-motifs and is dependent upon the polarity component *par6*.

### Effects of Fra-overexpression in epithelial cells

**Fig 2:**
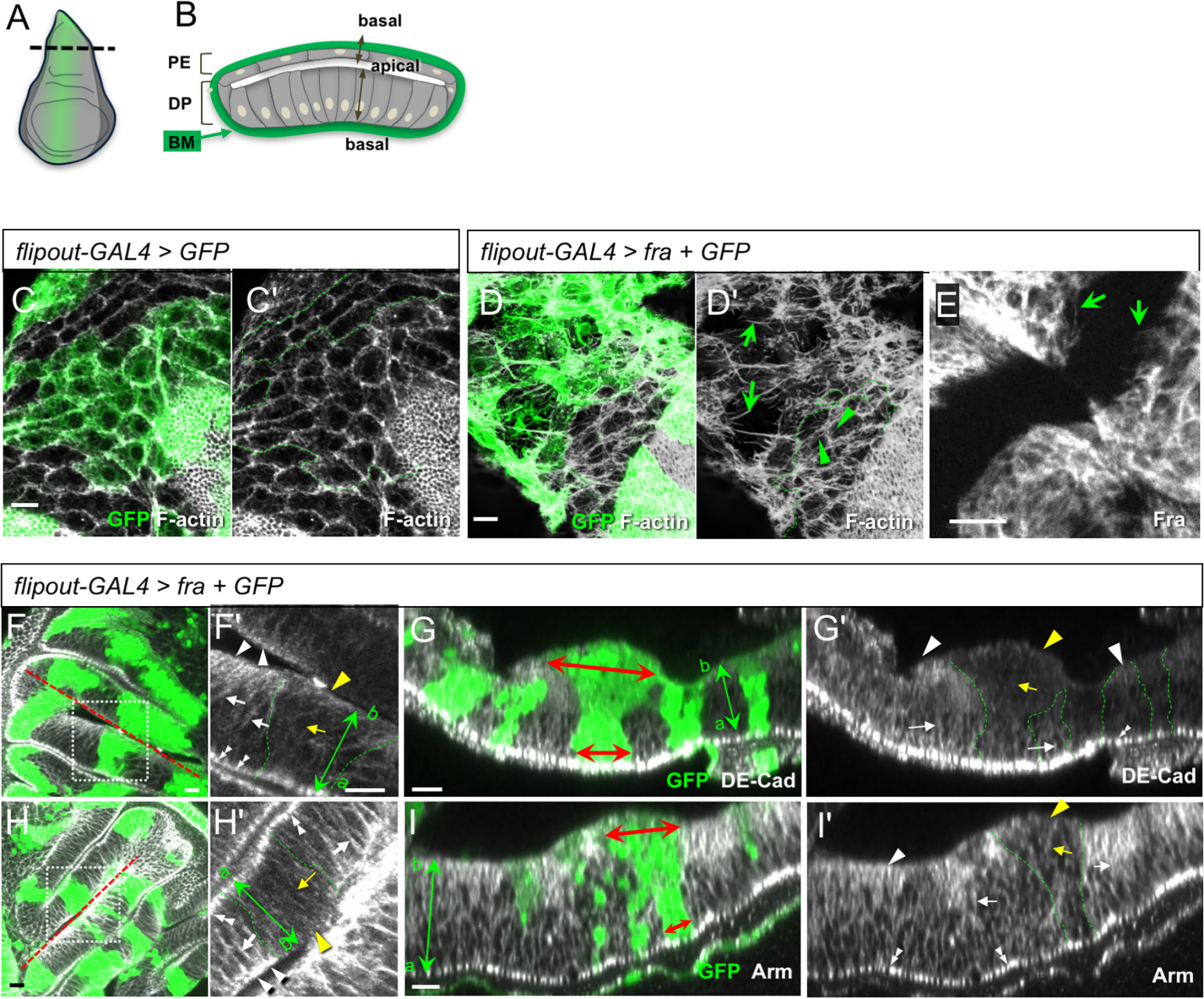
Effects of clonal overexpression of *fra* on F-Actin, cell shape and DE-Cad in epithelia. **(**A) Schematic of third instar wing disc. (B) Cross sectional view of A showing the squamous peripodial epithelium (PE) and the columnar disc proper epithelium (DP). The apical surfaces face the lumen while the basal surfaces face outwards and are covered in the basement membrane (BM, green). (C-I) Random *flipout-GAL4* clones expressing GFP (green) only (C) or GFP and *fra* (D-I) stained for F-Actin (C-D, grayscale), Fra (E), DE-Cad (F-G, grayscale), and Arm (H-I, grayscale), with GFP (green). (C) In control disc PE cells, F-actin is mainly enriched near the cell-cell junctions in both GFP-positive and GFP-negative clones. (D) In *fra*-overexpressing cells, F-actin filaments are often organized into thick extended bundles (arrows). In non-GFP wild type cells, F-actin filaments are also arranged in multiple shorter protrusions (arrowheads). Green dashed lines (C’, D’) indicate the borders of GFP-positive clones. (E) Expression of Fra induces protrusions in DP cells (arrows). (F-I) In DP epithelial cells, overexpression of Fra affects DE-Cad and Arm distribution and apico-basal cell shape. F' and H' show magnified view of boxed regions in F and H. G and I give cross-sectional view at dashed lines in F and H. In DP epithelial cells (F', G') DE-Cad localises to the zonula adherens (ZA) (double-arrowheads), basal (arrowheads) and lateral (arrows) sides. Cells overexpressing Fra (green) have reduced DE-Cad (F', G') and Arm (H', I') in basal (yellow arrowheads) and lateral (yellow arrows) regions and are expanded basally (red double-headed arrows, G, I). Genotypes: C: *hsFLP/+; Act5C*:*CD2*:*GAL4*, *UAS-GFP / +*, D-I: *hsFLP/+; Act5C*:*CD2*:*GAL4*,*UAS-GFP/UAS-fra*. Scale bars 10 μm.

Next, we wished to establish a range of cellular phenotypes in epithelia associated with *fra* overexpression. We created clones of epithelial cells overexpressing *fra*, using a mosaic flip-out GAL4 approach. As with *Ubx-GAL4*, mosaic GAL4 expression of Fra in the peripodial epithelium produced long protrusions that were rich in F-Actin (Fig 2D', arrows). Surprisingly, even in regions that were not over-expressing *fra*, extensive protrusions were observed (Fig 2D', arrowheads), a phenomenon never seen in control mosaic discs (Fig 2C'). Thus, *fra* overexpression elicits strong protrusive activity and, surprisingly, appears to have a non-autonomous effect on other cells in the epithelium.

Our mosaic analysis also revealed several phenotypes in disc-proper (DP) cells, an epithelial cell type that does not normally undergo EMT. Firstly, as with PE cells, DP cells extended motile protrusions, which occurred in basal regions of the cell (Fig 2E). Clones of DP cells also exhibited a change in cell shape whereby they were expanded on the basal side (Fig 2G' and 2I', red double-headed arrows). DP cells also showed a change in AJ component distribution. In control cells, DE-Cad was strongly expressed at the zonula adherens (ZA) (Fig 2F', double-arrowheads), but was also found in puncta that were densely accumulated in basal regions of the cell (Fig 2F', arrowheads), and at lower levels in the middle of the lateral membrane (Fig 2F', arrows). In *fra* expressing clones, DE-Cad was reduced in basal regions and also slightly decreased laterally (Fig 2F' and 2G', yellow arrows and arrowheads). A similar reduction in Arm expression in basolateral regions of DP cells was also seen (Fig 2H' and 2I'). DE-Cad and Arm levels at the ZA did not appear to be altered though it is possible that the intensity of expression at ZAs would obscure subtle changes. In PE cells, changes in DE-Cad expression along the apico-basal axis could not be determined due to the squamous morphology of these cells, but ZA associated DE-Cad appeared normal (data not shown).

Thus, overexpression of Fra in epithelial cells has three effects: extension of protrusions, an apico-basal cell shape change, and mislocalization of AJ components.

### Fra induces motile protrusions, which are dependent upon the P3 motif

**Fig 3:**
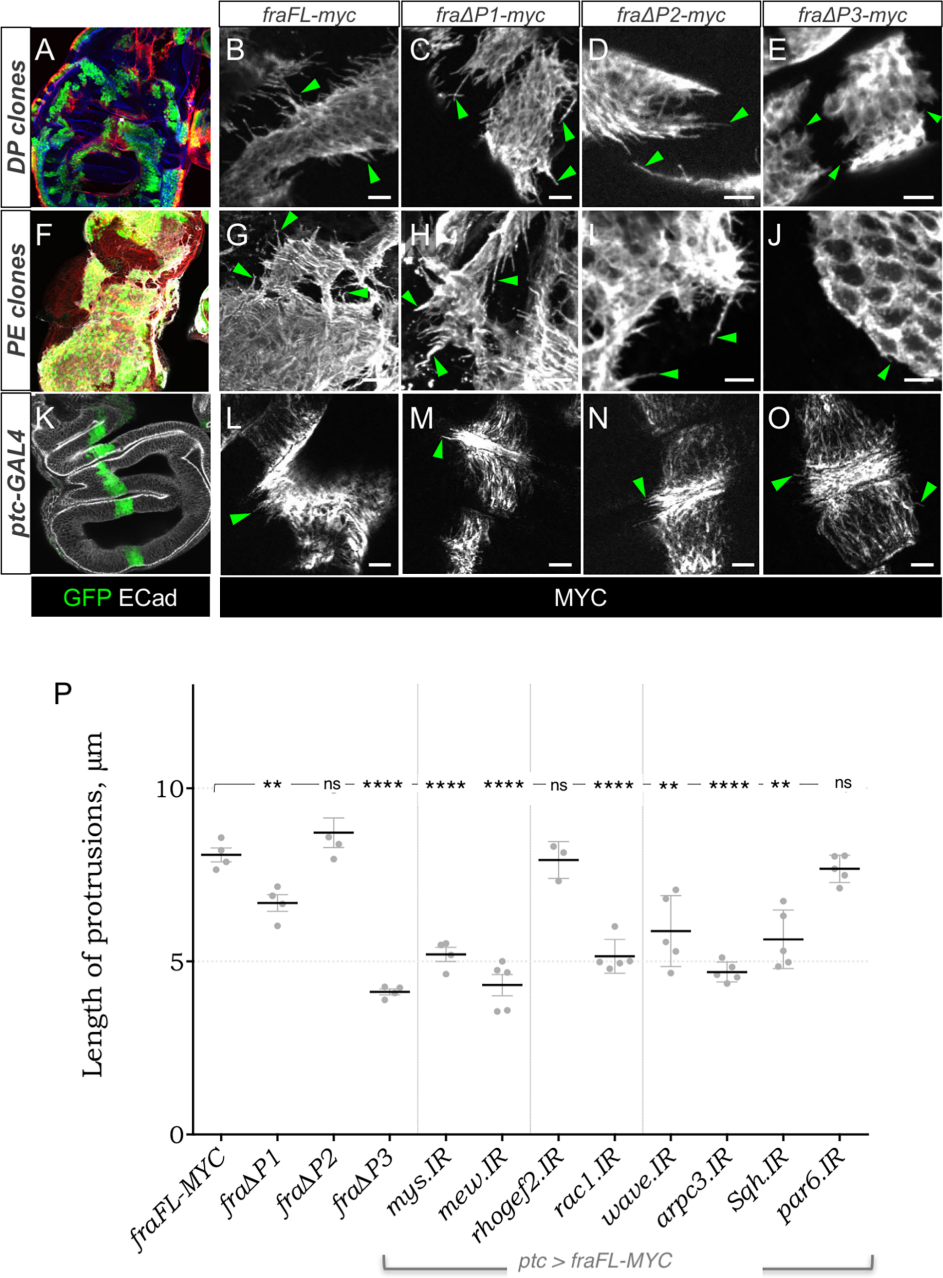
Overexpression of *fra* induces motile protrusions. Mosaic *hsFLP;Act5C*:*CD2*:*GAL4*,*UAS-GFP* (A-J) or *ptc-GAL4* (K-O) wing discs expressing *fra-*deletion transgenes and stained for Myc (grayscale). A full-length Fra transgene induces long protrusions (arrowheads) in both DP (B, L) and peripodial cells (G). Fra transgenes lacking either the P1 (C, H, M) or P2 (D, I, N) motifs elicited protrusions of similar length to full-length *fra*, but *fra*∆*P3*-expressing cells extended much shorter protrusions (E, J, O, arrowheads). (P) Quantification of length of protrusions in *ptc-GAL4* wing discs expressing the indicated *fra* transgene. Deletion of the P1 motif has a mild effect on length, while loss of the P3 motif halves the length of protrusions. Knockdown of other genes modifies protrusion length caused by *fraFL-myc* (see text for details). Note that expression levels of the four transgenes appeared similar (immunostaining and imaging parameters were kept consistent across genotypes). Error bars show mean ± SEM of multiple discs. Significance values based on two-tailed students t-test: ns not significant, ** p-val<0.01, **** pval<0.0001. Scale bars 10 μm.

Next, we wished to determine which P-motifs were required for these three phenotypes. To compare the effects of different *fra* transgenes, quantification was needed, but this was difficult in mosaic discs, due to the random size and location of clones. We therefore used the *ptc-GAL4* driver, which expresses in a consistent narrow stripe between the anterior and posterior compartments in the DP epithelium (Fig 3K).

First, we compared the ability of ∆P transgenes to create protrusions in both the PE and DP epithelia using mosaic expression. Protrusions were clearly visualised with anti-Myc staining indicating that Fra-Myc localised to the membrane of these fine cellular processes. As previously reported [23], all transgenes expressed at similar levels. Protrusions in both PE and DP cells were substantially reduced in *ptc>fra∆P3-myc* discs (Fig 3E and 3J), and slightly reduced in *ptc>fra∆P1-myc* discs (Fig 3C and 3H), while those in *ptc>fra∆P2-myc* wing discs appeared similar to controls (Fig 3D and 3I).

Next we quantified these observations with the *ptc* driver. *ptc-GAL4* expression of *fraFL-myc*, induced basal protrusions in DP cells, which emerged from the edges of the *ptc* domain (Fig 3L) and had mean length of 8.08 ± 0.2 μm (SEM) (Fig 3L and 3P). As with mosaic clones, protrusions were substantially reduced in *ptc>fra∆P3-myc*, discs down to 4.11 ± 0.09 μm (p < 0.0001; Fig 3O and 3P) and somewhat reduced in *ptc>fra∆P1-myc* discs to 6.69 ± 0.24 (p = 0.0046) (Fig 3M and 3P), while those in *ptc>fra∆P2-myc* wing discs were not significantly different from controls (8.72 ± 0.43; p>0.05) (Fig 3N and 3P). Thus, the P3 motif is critical for protrusions, while the P1 motif plays a lesser role, and the P2 motif is not required.

Given the basal location of these protrusions we speculated that they might be adhering to the basement membrane (BM) via integrins. Consistent with this, RNAi knockdown of *mys/beta-PS* and *mew/alpha-PS1* in *ptc>fraFL-myc* discs reduced protrusion length to 5.2 ± 0.2 μm and 4.3 ± 0.3 μm respectively (p<0.0001 for both). In addition, knockdown of *Rac1*, its effector *wave* and the Arp2/3 component *arpc3*, as well as the myosin, *Sqh*, all significantly reduced protrusion length (Fig 3P). The effects of *Rho1* knockdown were also tested but could not be quantified due to drastic changes in the morphology and distribution of *ptc>GFP* cells (see below). As an alternative way of modulating Rho1 pathways we tested whether *rhogef2* could influence protrusions but it had no effect.

The results suggest that Fra activates a typical axon-guidance motility pathway in DP cells, in which Rac1 promotes F-Actin-rich protrusions, which adhere to the BM.

### Fra causes basal expansion in disc proper cells, which is dependent upon the P1 motif

**Fig 4:**
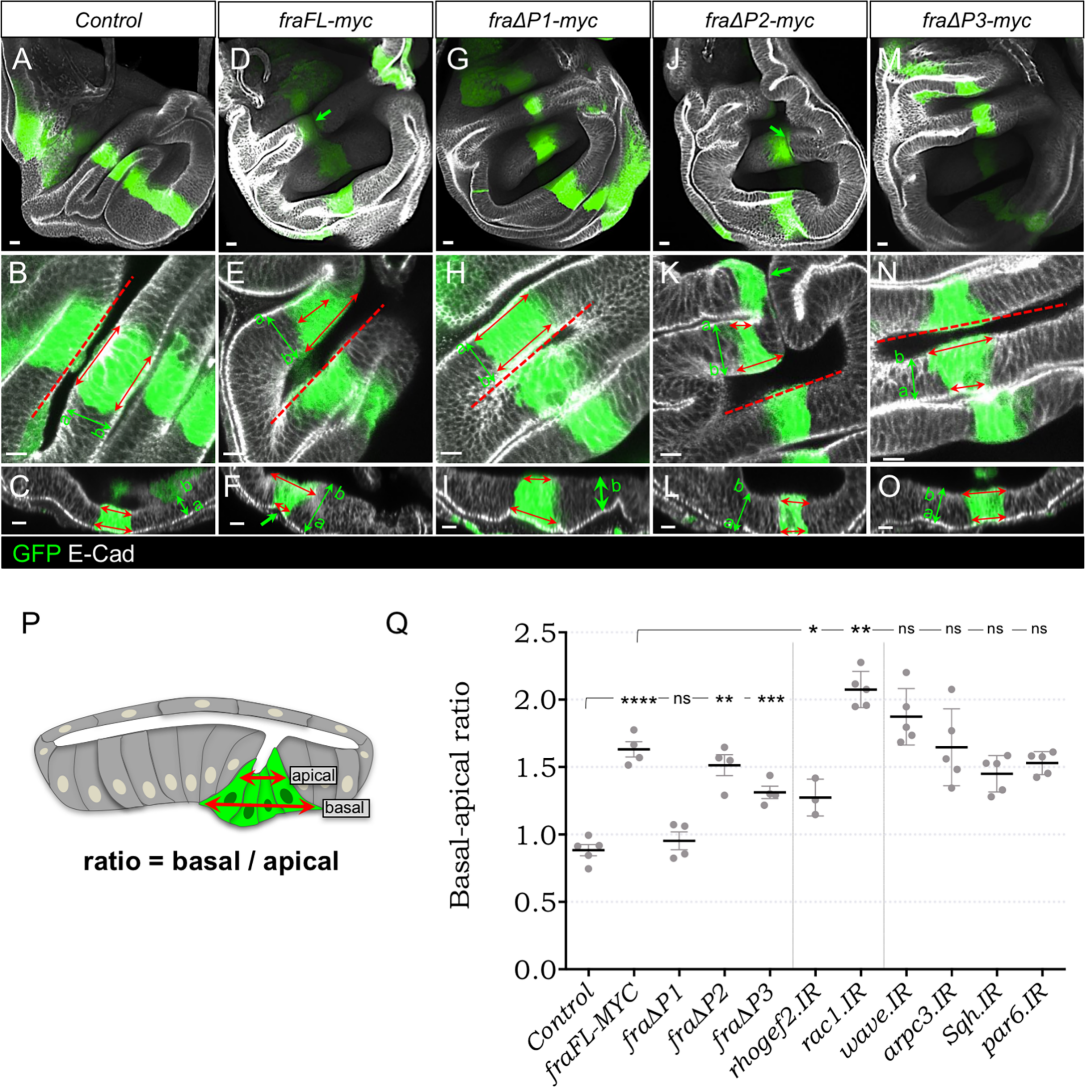
Overexpression of *fra* induces basal expansion of the DP epithelium. *ptc>GFP* wing discs stained for GFP (green) and DE-Cad (grayscale). Red dashed lines (B, E, H, K, N) indicate position of cross sections depicted in (C, F, I, L, O). (A) control disc showing the expression domain of ptc-GAL4. (B, C) DP cells are roughly columnar: i.e. the lateral extent of the *ptc-GAL4* domain in apical and basal parts of the cells (red double-headed arrows) is similar. (D) Expression of *fraFL-myc* creates a fold through the disc (D, F, arrow). (E-F). The *ptc-GAL4* domain is wider in basal regions. (G-I) Expression of *fra∆P1-myc* is unable to create the apical fold or change in aspect ratio. (J-L) *fra∆P2-myc* is as effective as full-length Fra in generating folds and shape change. (M-O) *fra∆P3-myc* affects the shape change to an intermediate degree, but did not create folds. (P) Schematic showing method for quantification. (Q) Quantification of basal-apical ratio in *ptc-GAL4* wing discs expressing the indicated *fra* transgenes. (see text and for details). Error bars show mean ± SEM of multiple discs. Significance values based on two-tailed students t-test: ns not significant, ** p-val<0.01, **** pval<0.0001. Scale bars 10 μm.

As with mosaic discs (Fig 2G and 2I), *ptc>fraFL-myc* discs also exhibited a cell shape change, whereby the basal side of cells was expanded and the apical side reduced (Fig 4E). In addition, there was a furrow generated along the whole *ptc* stripe (Fig 4D). To quantify the cell shape change we calculated the ratio between basal and apical sides of epithelial cells within the *ptc-GAL4* expression stripe (Fig 4P). In control *ptc>GFP* wing discs, GFP-positive cells had either a rectangular shape or were slightly constricted on the basal side (mean ratio = 0.88 ± 0.04) (Fig 4A–4C and 4Q). In *ptc>fraFL-myc*,*GFP* cells the apical/basal ratio was significantly increased to 1.63 ± 0.06 (p = 0.0009; Fig 4E, 4F and 4Q).

We next tested which P-motifs were required for the apico-basal cell shape change, again using the *ptc-GAL4* driver. In *ptc>fra∆P2-myc* discs the apical-basal ratio was 1.514 ± 0.08 similar to *ptc>fraFL-myc* discs (p = 0.38, Fig 4J–4L and 4Q). Expression of *fra∆P3-myc* produced an intermediate effect in which the basal-apical ratio was increased to 1.31 ± 0.045, which was significantly higher than in controls (p = 0.0002) but also significantly lower than in *ptc>fraFL-myc* wing discs (p = 0.0048) (Fig 4M–4O and 4Q), though no apical furrows were detected. In contrast, expression of *fra∆P1-myc* had no effect on shape change. The ratio of 0.95 ± 0.06 was not significantly different from the *ptc>GFP* control (p>0.3; Fig 4G–4I and 4Q).

These changes in cell shape could potentially be due to effects on contractility (e.g. either a decrease in basal contractility or an increase in apical contractility). Alternatively, the basal protrusions could cause apical furrows by adhering to the BM and generating a traction force that pulls the epithelium down. In support of the first model we found that cell-shape changes were suppressed by knockdown of *rhogef2* (ratio = 1.27 ± 0.08; p = 0.013, Fig 4Q) and actually enhanced by knockdown of *Rac1* (ratio = 2.075 ± 0.06; p = 0.0012, Fig 4Q).

**Fig 5:**
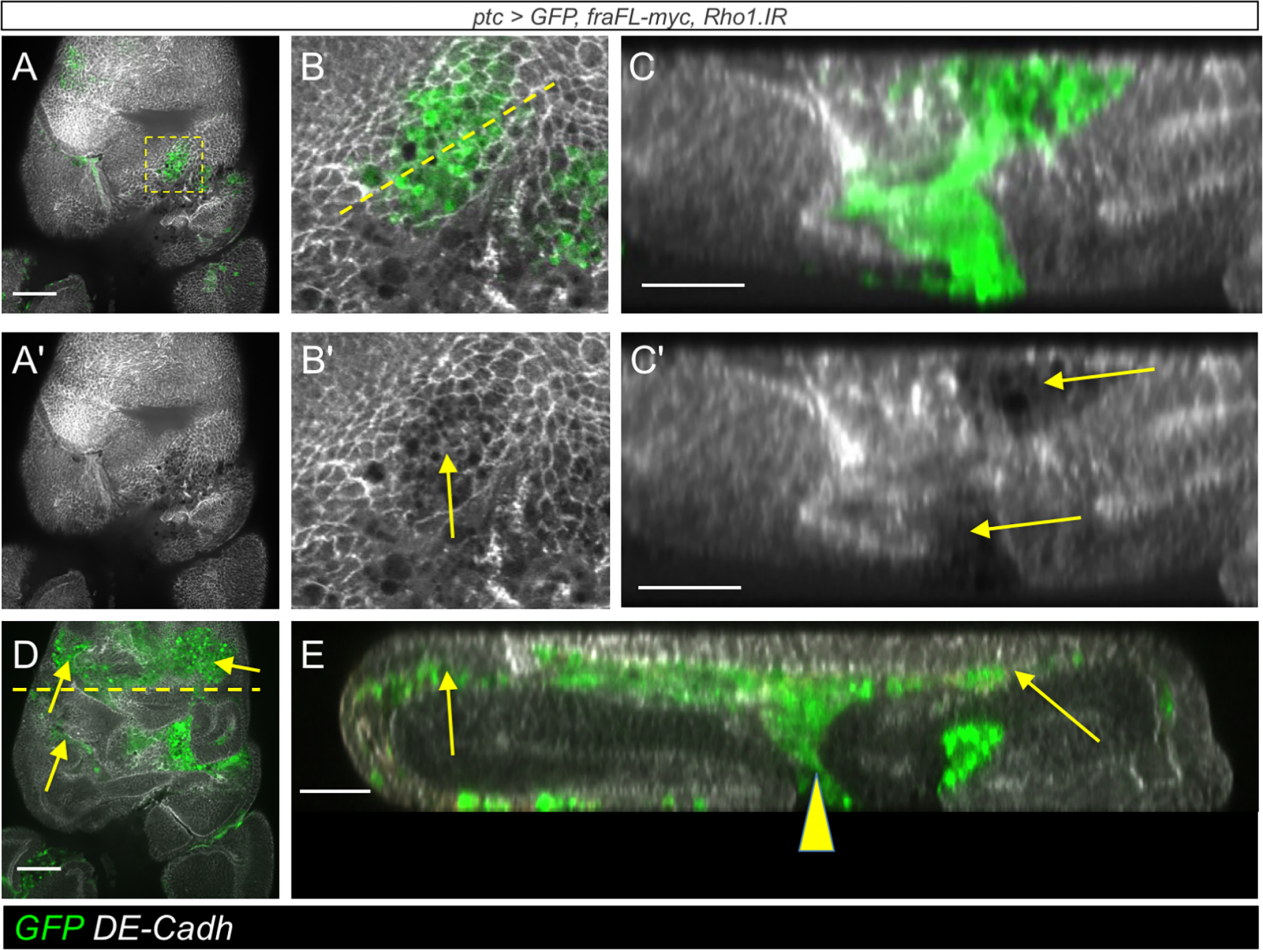
RNAi knockdown of *Rho1* in *fra*-expressing cells disrupts epithelial integrity. (A-E) *ptc>GFP*, *fraFL-myc*, *Rho1*.*IR* disc stained for DE-Cad (grayscale) with GFP (green). (A) Section of the disc at the level of the apical surfaces of the DP and PE epithelia. (B) Close up of the boxed region in A. Cells in the GFP-expressing domain do not have the normal ZA cell-cell junctional staining and levels of DE-Cad are reduced (B', arrow). (C) Cross-section at the position of the dashed line in B. GFP expressing cells show a strong reduction in DE-Cad levels (C', arrows). (D) More basal view of the same disc showing GFP expressing cells have disseminated away from the central ptc-GAL4 stripe (arrows). (E) Cross section at the position of the dashed line in D. GFP cells have spread out from the central GFP stripe area (arrowhead) and disseminated in the space between the apical surfaces of the PE and DP epithelia (arrows). Scale bars 50 μm (A, D), 20 μm (C, E).

To show the contraction was dependent upon Rho1, we attempted to rescue the phenotype by co-expression of *UAS-Rho1*.*IR*. However, *ptc>fraFL-myc*, *Rho1*.*IR* discs were too highly disrupted to perform the quantification. Nevertheless, it was clear that GFP-expressing cells had reduced levels of DE-Cad and loss of ZAs, and had broken free of the epithelium and disseminated throughout the lumenal space between the apical surfaces of the DP and PE (Fig 5). Thus, in Fra-overexpressing cells, Rho1 appears critical for maintenance of epithelial integrity.

**Fig 6:**
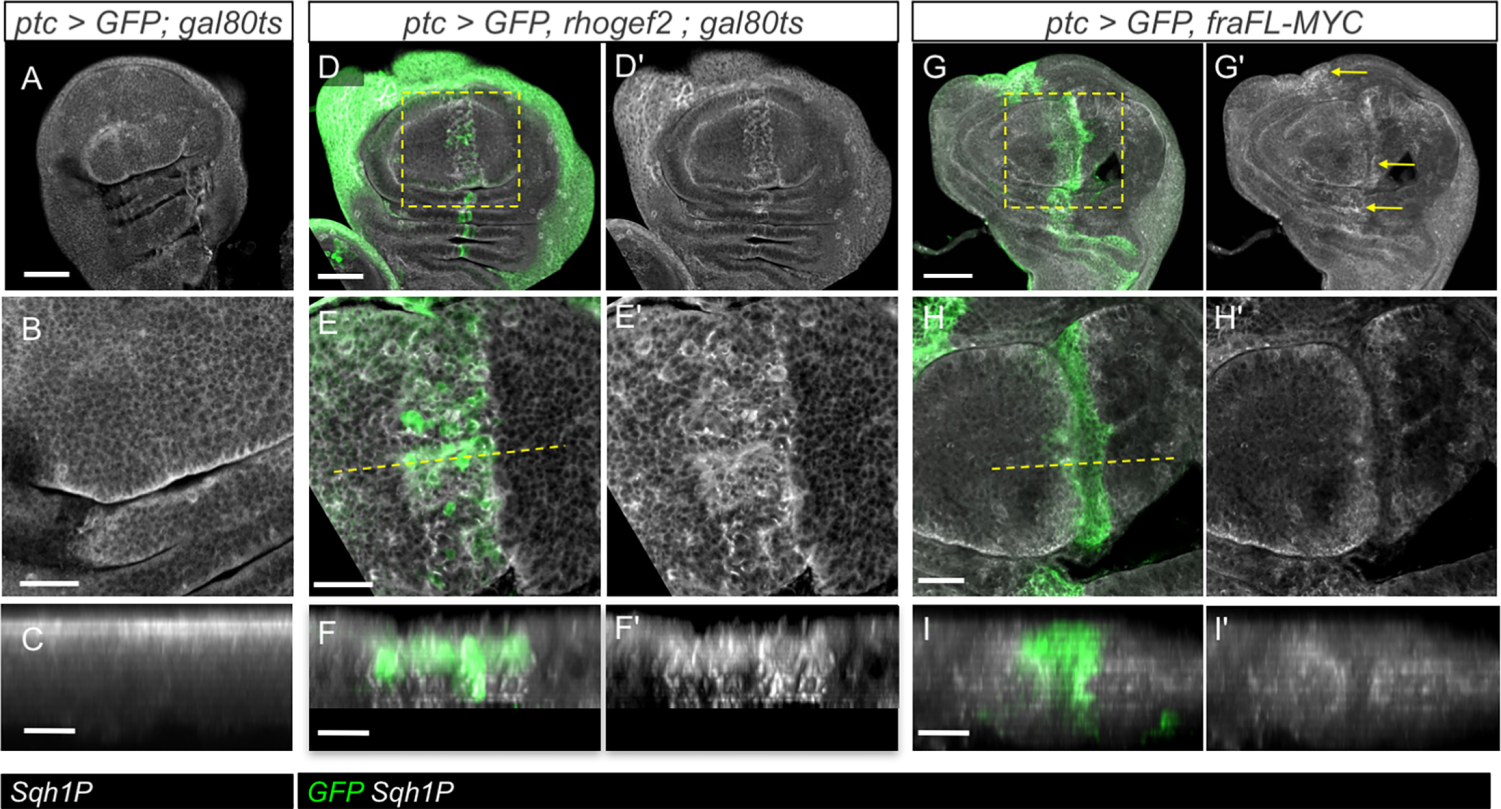
Overexpression of *fra* activates a Rho1 contractility pathway. (A-C) Control *ptc-GAL4*,*UAS-GFP;gal80*^*ts*^ wing disc stained for phosphorylated myosin regulatory light chain (pSqh1P, grayscale). Active Sqh outlines cells, and is relatively constant across the wing pouch area. (D-F) Expression of *rhoGEF2* for a period of 4 hours causes furrowing in the *ptc-GAL4* domain accompanied by increased levels of Sqh1P. (G-I) Expression of *fraFL-myc* causes furrowing and a band of increased levels of Sqh1P along the *ptc-GAL4* domain (G', arrows). Scale bars 50 μm (A, D, G), 20 μm (B, E, H), 10 μm (C, F, I).

To further test the idea that Fra-overexpression was activating an apical contractile Rho1-pathway we utilised the Sqh1P antibody [36], which detects an active, phosphorylated form of the myosin Spaghetti Squash (Sqh) (Fig 6). We first tested the antibody on wing discs in which a furrow was induced using a *UAS-rhogef2* transgene, which is known to activate Rho1-pathways. Since *ptc>rhogef2* discs were very strongly folded we temporally restricted the period of expression using a *ptc-GAL4*, *gal80*^*ts*^ driver. Expression of *rhogef2* for 4 hrs at 29°C caused a mild furrow which was clearly enriched for Sqh1P (Fig 6D' and 6E'). In *ptc>fraFL-myc* discs a similar, though milder, increase in Sqh1P levels in the vicinity of the furrow was seen (Fig 6G', 6H' and 6I').

The results are consistent with a model in which Fra activates a Rho1-contractile pathway which causes apical constriction and a change in the apico-basal ratio, and this is mediated primarily through its P1 domain. Thus, protrusions and shape change are molecularly separable both in terms of the domains of Fra required and the downstream genes involved.

### Overexpression of full-length *frazzled* reduces levels of DE-Cad in the basolateral regions of disc proper cells

**Fig 7:**
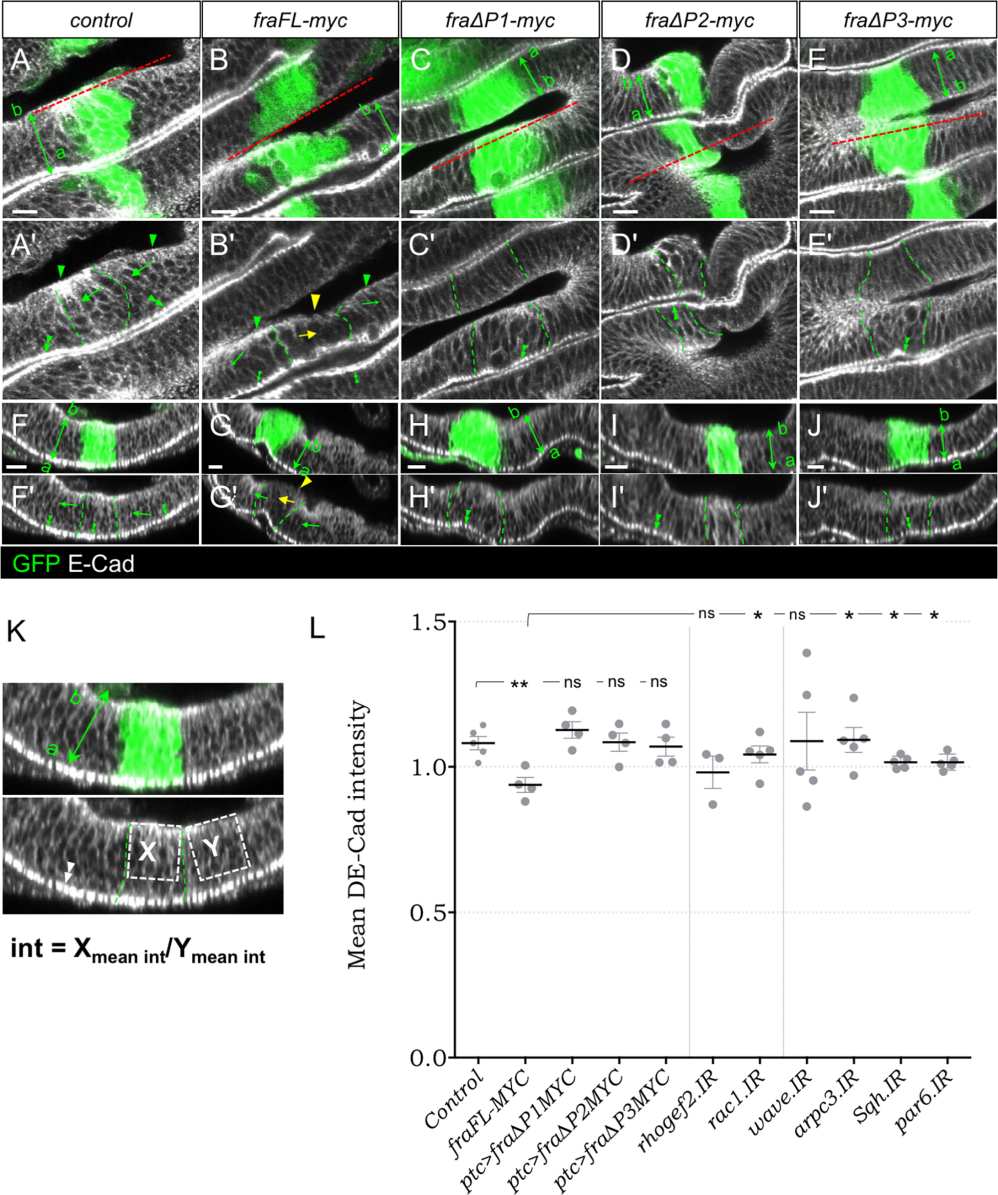
Overexpression of *fra* reduces DE-Cad in basolateral regions of the DP epithelium. *ptc>fra*,*GFP* wing discs (GFP shown in green) immunostained for DE-Cad (grayscale). Dashed lines (A-E, red) indicate region shown in cross-sections (F-J). (A) In control discs, DE-Cad is strongly localised to the ZA (A’, F’, double-arrowheads), and on the basal (arrowheads) and lateral (arrows) sides of DP cells. (B) Expression of *fraFL-myc* significantly reduces DE-Cad in the basal (yellow arrowhead) and lateral sides (yellow arrows). (C-J) Expression of the *fra∆P-myc* transgenes does not affect DE-Cad localization. (K) Quantification of change in mean basolateral DE-Cad intensity. (L) Mean intensity of DE-Cad in *ptc*-GFP domain relative to adjacent non-GFP areas in wing discs for different genotypes. Error bars show mean ± SEM of multiple discs. Significance values based on two-tailed students t-test: ns not significant, * p-val<0.05, ** pval<0.01. Scale bars 10 μm.

Next, we again used the *ptc-GAL4* driver to confirm and quantify the reduction of basal DE-Cad previously seen in mosaic flip-out discs. Overexpression of *fraFL-myc* with *ptc-GAL4* caused a significant loss of DE-Cad from basal and lateral sides of the DP with the ratio changing from 1.08 ± 0.023 in control *ptc>GFP* discs to 0.94 ± 0.026 in *ptc>fraFL-myc*, *GFP* discs (p = 0.0043; Fig 7B' and 7L). DE-Cad loss from basolateral regions was also partly dependent upon *Rac1* and *arpc3* suggesting that this phenotype may be linked to the molecular pathways controlling F-Actin protrusions (Fig 7L). Finally, we tested the ∆P transgenes. However, none of the *fra* ∆P deletion transgenes could significantly alter DE-Cad distribution (p>0.2; Fig 7C–7E, 7H–7J and 7L). Since only full-length Fra could cause mislocalization of DE-Cad, it suggests that this phenotype is either distinct from the Rac1 and Rho1 molecular pathways, or is dependent upon both.

### Fra-dependent inhibition of eversion and loss of basolateral DE-Cad requires *par6*

**Fig 8:**
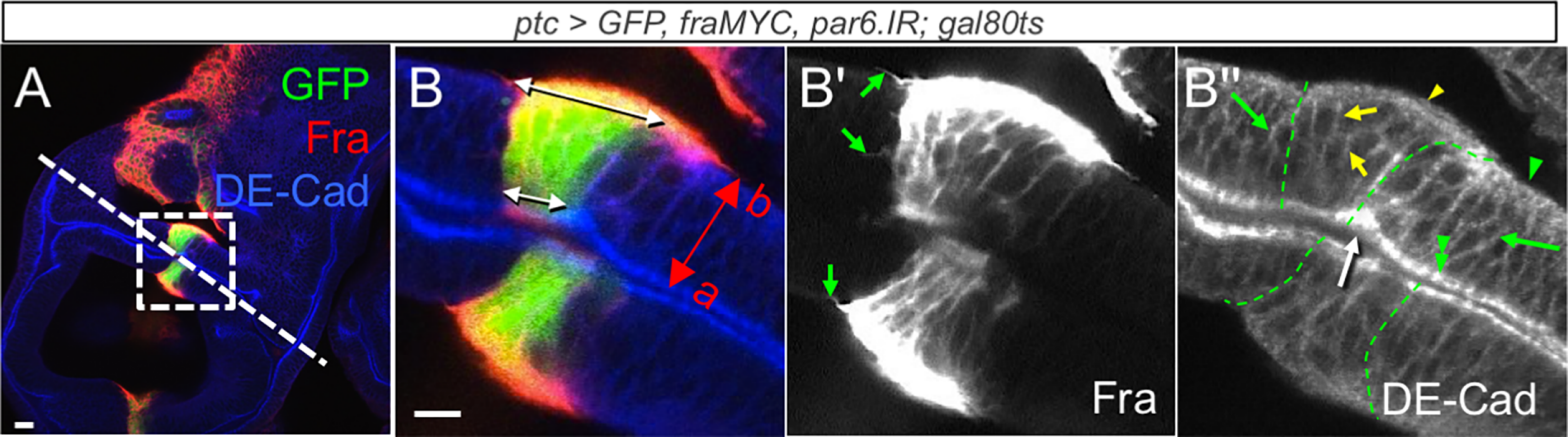
*par6* knockdown rescues *fra*-associated mislocalisation of DE-Cad. (A, B) A *ptc>fraFL-myc*, *par6*.*IR*, *GFP* wing disc immunostained for Fra and DE-Cad. (A) DP of *ptc>fraFL-myc*, *par6*.*IR*, *GFP* disc. Dashed boxes indicate magnified area depicted in B. (B) The *ptc>fraFL-myc*, *par6*.*IR* cells display typical basal expansion (double-headed arrows, B) and basal protrusions (arrows, B'). The intensity of basolateral DE-Cad in *ptc>FraFL-myc*, *par6*.*IR* cells (yellow arrowhead and yellow arrows, B'') is comparable with wild type cells (green arrowhead and green arrows, B'')). ZA are indicated by double arrowheads (B''). A furrow (white arrow) also formed along the GFP-positive (green) stripe. Scale bars 10 μm.

Finally, given the importance of *par6* in blocking eversion we tested whether it was required for the epithelial cellular phenotypes. *par6* knockdown partially rescued the loss of basolateral DE-Cad but not the shape change and protrusions. *ptc>fraFL-myc*, *par6-IR* discs exhibited an intermediate level of basolateral DE-Cad (Figs 8B and 7L): the mean intensity (1.01 ± 0.012) was significantly higher than in *ptc>fraFL-myc* cells (0.94 ± 0.026, p = 0.022), but lower than in control *ptc>GFP* cells (1.08 ± 0.023, p = 0.036). In contrast, there was no significant difference from controls in the average length of protrusions (7.7 ± 1.98 μm, p >0.7, Figs 8B' and 3P), or the basal-apical ratio (1.53 ± 0.26, p >0.3, Figs 8B and 4Q).

Thus, *par6* is partially required for adult eversion defects and DE-Cad localization, but not for shape-change or protrusions. This suggests that the DE-Cad phenotype, and not the protrusions or shape change, lie at the heart of the inhibition of EMT, and that regulation of DE-Cad localization is molecularly separable from the Rho/Rac *fra*-induced pathways controlling shape change and protrusions.

## Discussion

### Frazzled regulates cellular protrusions

Although the signaling pathways acting downstream of DCC-family receptors in growth cones and migrating cells are well studied, little is known about those operating in epithelial cells. In this study, we have presented evidence that the ability of Fra to generate motile protrusions, is molecularly separable from its ability to modulate epithelial cell shape and AJ component localization, in that each of these responses has characteristic requirements for the Fra intracellular motifs, and for downstream genes.

Frazzled expression in both the PE and DP epithelia induced extensive cellular protrusions which were enriched for both F-Actin and Frazzled. The ability of a DCC-family receptor to promote F-Actin structures in non-neural cell types has previously been reported for epithelial cell lines [37,38], fibroblasts [39], and stem cells [40]. In neurons, DCC-family receptors promote F-Actin protrusions via activation of Rac1 and/or Cdc42 [30,37,39,41]. Our results suggest epithelial cells can activate a similar pathway as protrusions were dependent upon *Rac1*, its downstream effector *wave*, as well as *arpc3*, a component of the Arp2/3 F-Actin polymerization complex. The integrins *mys/beta-PS* and *mew/alpha-PS1* were also required suggesting that, like axons, epithelial protrusions adhered to the underlying ECM. The protrusions were also critically dependent upon the P3 motif, which again is consistent with axon guidance studies. The P3 motif has also been shown to activate non-muscle myosin, and, consistent with this, we found protrusions depended on the myosin *sqh*. The P1 motif was also partly required for protrusion formation which could indicate a role for the actin filament elongation factor Ena, as demonstrated for UNC40 [30] but we were unable to test the role of *ena* due to early lethality.

### Frazzled regulates cell shape change

Overexpression of Frazzled also caused a cell shape change in which the normally columnar DP cells exhibited a basal expansion and apical constriction, accompanied by an apical furrow. These sorts of morphological changes, which are often associated with tissue invagination processes, typically involve activation of a Rho1 pathway in apical regions of the cell leading to recruitment and activation of actin polymerization factors, such as the formin Diaphanous, and the contractile, non-muscle myosin II [42,43]. Consistent with this model, the shape changes were dependent upon RhoGEF2, and ectopic expression of RhoGEF2 could elicit similar contractile furrows to Fra. Furthermore, Fra expression resulted in an increase in phosphorylated Sqh. Strikingly, these shape changes were actually increased by knockdown of *Rac1*. This rules out a model in which adhesion of the protrusions on the BM "drags down" the epithelium, but rather suggests that Rac1 is inhibiting shape change, perhaps by antagonising the activity of Rho1, as occurs in other systems [44,45].

It is also likely that activation of a contractile pathway inhibits the peripodial EMT given the central role Rho1 pathways play in maintaining stable cell-cell junctions [46]. For example, in MDCK cells AJs are strengthened by E-Cadherin enrichment in response to elevated contractility [47]. A direct test of Rho1 involvement in Fra-induced shape changes was complicated by the strong effect of *rho1* RNAi, whereby ZA and epithelial integrity were greatly disrupted. Nevertheless, the results suggest that Rho1 plays a crucial role in maintaining ZA stability in Fra-overexpressing cells, presumably by increasing apical contraction.

Shape changes were also strongly dependent upon the P1 motif. Given the P1 motif is not required for axonal midline guidance decisions [23] this suggests a distinct P1-dependent and Rho-dependent pathway is being activated by Fra in epithelial cells. How Frazzled might promote Rho1 activation is unclear since in mammalian neurons, Netrin-1/DCC signal transduction actually inhibits RhoA [39]. In *Drosophila*, Fra has been shown to act synergistically with active Rho1 and myosin activation, but this interaction required the P3 motif and appeared to be inhibited by the P1 motif [29]. Thus, further work will be required to understand the link between the P1 motif and Rho1 pathways.

### Frazzled regulates DE-Cad localization

Fra overexpression also caused a reduction in basolateral DE-Cad. Loss of DE-Cad from the basolateral membrane may be of key importance in the ability of Fra to inhibit eversion for two reasons. Firstly, only full-length Fra could efficiently block eversion and DE-Cad delocalization was the only cellular phenotype that required full-length Fra. Secondly, *par6*, which appears to play a key role in eversion failure, was only required for the DE-Cad delocalization phenotype.

In normal epithelial cells, DE-Cad undergoes extensive trafficking and turnover, which helps maintain intercellular adhesion during morphological changes such as tissue invagination, EMTs, cell intercalation and cell extrusion (for reviews see [48,49]). In mammalian cells, newly synthesized E-Cad from the Golgi, fuses with Rab11 recycling endosomes, and is subsequently trafficked to the basolateral membrane [50]. Similarly, in *Drosophila* follicular epithelial cells, Rab11 is required for DE-Cad trafficking to the membrane [51]. This latter study proposed a Rab11-dependent apicolateral trafficking pathway and a Rab11-independent lateral pathway. DE-Cad vesicles from the recycling endosome may be specifically targeted to regions of the membrane containing AJ components since the exocyst component Sec15 can directly interact with Arm [52]. Rho1 is also thought to play a role in the formation of Rab11-dependent recycling endosomes, which pinch off from the common recycling endosome [53]. Once in the lateral membrane, DE-Cad can move to the ZA region via at least two routes. Firstly, it can be endocytosed and then recycled to the ZA region via a Rab11/RabX1 pathway [51]. This internalization of DE-Cad is dependent upon Rab5 and upon the Arp2/3 complex, which acts downstream of the Cdc42-aPKC-par6 apical complex [54,55]. Secondly, it can undergo a non-vesicle based, basal-to-apical flow, which is dependent upon cortical F-Actin [51,56].

Therefore, Fra could potentially cause a reduction in basolateral DE-Cad by disrupting the balance between one or more of these processes. For example, Fra may inhibit delivery of DE-Cad to the lateral membrane by inhibiting Rab11. This idea is supported by the similarity in phenotypes between *rab11* mutants and Fra-overexpressing cells. In the *Drosophila* follicular epithelium in *rab11* mutants there is a loss of apico-lateral exocytosis of DE-Cad but no significant changes in ZA structure [51]. Unfortunately, a direct test of the importance of Rab11 in the Fra phenotypes as not possible due to high larval lethality associated with *ptc-GAL4* expression of *UAS-rab11*.*IR*. Redistribution of DE-Cad could also be caused by changes in Rho GTPase activity. For example, Rho1 can increase the density of Rab11 positive vesicles at the level of the apical ZA and may therefore increase delivery to more apical regions [53]. Similarly, the extensive Rac1-dependent changes in F-Actin in more basal parts of Fra-expressing cells could potentially influence both the endocytosis process and the basal-apical flow.

Finally, *par6* was also required for Fra-dependent loss of basolateral DE-Cad and for blocking adult eversion. Par6 is known to act with Cdc42 and aPKC in promoting the actin-dependent endocytosis of AJ components [54,55]. Given Neogenin can promote E-Cad endocytosis in human intestinal epithelial cells [20] it may be that Fra is similarly increasing DE-Cad internalization and, thereby, a reduction in lateral DE-Cad.

In summary, Fra expression in epithelial cells has a range of effects which involve distinct molecular pathways. Our data suggest that the ability of Fra to promote epithelial stability is likely due to par6-dependent regulation of DE-Cad trafficking but further work will be required to test this hypothesis. It will now be important now to examine the role of these molecular pathways in the EMT/MET events that Fra regulates.

## Materials and methods

### Drosophila stocks

The following fly strains were used: *w*^*1118*^ (Bloomington); *Ubx-GAL4* (a kind gift from L. S. Shashidhara [57]); *UAS-fra-myc*, *UAS-fra∆P1-myc*, *UAS-fra∆P2-myc*, *UAS-fra∆P3-myc* (a gift from G. J. Bashaw [23]); *patched-GAL4*, *UAS-mCD8-GFP*, *UAS-fra*, *UAS-dicer2*, *UAS-GAL4* and *Df(2R)BSC880* were obtained from the Bloomington Drosophila Stock Centre; *hsFLP Act5C-FRT-CD2-FRT-GAL4*, *UAS-GFP* and *hsFLP; Act5C-FRT-CD2-FRT-GAL4*, *UAS-RFP* a gift from T. Brumby). *UAS-rhoGef2* (a gift from H. Richardson). All UAS-RNAi -stocks that were used for wing disc eversion assay or in epistasis test were obtained from either the Vienna Drosophila Resource Centre (VDRC) or Bloomington Drosophila Stock Centre.

### Immunostaining of imaginal discs

The following antibodies were used: DCAD2 (rat-anti-DE-Cad) used at 1/200 and Mab N27A1 (mouse-anti-Armadillo) used at 1/200 (Developmental Studies Hybridoma Bank); Rabbit-anti-Frazzled: a kind gift from Florence Maschat [4] used at 1/500; Rabbit-anti-GFP used at 1/500 (Life Technologies). Guinea-pig anti-Sqh1P [36] a kind gift from Robert E. Ward IV used at 1/100. To label F-actin, tissues were incubated with 50 μM Rhodamine-conjugated phalloidin (or alternately Alexa-488 Phalloidin or Alexa-555 Phalloidin; Invitrogen) in PBS+0.1% Triton X-100 (hereafter PBS-T).

Wing imaginal discs were dissected out of wandering third instar larvae in PBS, and fixed in 3.7% formaldehyde in PBS for 15 minutes. The discs were then washed three times in PBS-T, each wash for 15 minutes. Primary antibodies diluted in PBS-T were incubated with the discs for 3 hours at 25°C or overnight at 4°C. The incubation with the primary antibodies could be combined with phalloidin. Then, discs were washed three times in PBS-T quickly and then four more times, each for 15 minutes, incubated with secondary antibodies in PBS-T for 2 hours at 25°C or overnight at 4°C, washed again in PBS-T three times quickly and then four more times, each for 15 minutes, then cleared in 70% glycerol in PBS and mounted for imaging. The mounted discs were then imaged using confocal microscopy.

### Mosaic wing discs and heat-shocking

To create clones in flip-out wing discs, flies were raised in vials at 25°C for 72 ± 11 hours after setting up the cross, at which point larvae were roughly first instar stage. Then, the adults were removed, and the vial was placed in a water bath incubator at 37°C for 15 minutes. Wandering third instar larvae were dissected in PBS and mosaic wing imaginal discs used for further in vitro culture or immunostaining.

### Microscopy and image analysis

Fluorescence microscopy was performed on an Olympus FV1000 confocal microscope. All images acquired were at 1024-pixel resolution. To quantify protrusions, the length of the DP protrusions in the hinge region was measured in the *ptc-GAL4* area of wing discs expressing myc-tagged *frazzled* transgenes. For each disc, at least 10 protrusions were measured, and a single average value obtained per disc. To quantify basal expansion, the ratio between basal and apical sides of epithelial cells within the *ptc-GAL4* expression region in the DP epithelium was calculated (Fig 6P). To quantify the levels of DE-Cad immunostaining for a disc we calculated the ratio of mean intensity (arbitrary units) between a *ptc-GAL4* expression region and an adjacent non-*ptc* region of the DP epithelium (Fig 7K). Basal expansion and DE-Cad intensity measurements were performed on the cross-sectional slices in the hinge region of the *ptc-GAL4* expression stripe. A single value for each disc was obtained by taking the average of 4–7 measurements. ImageJ was used for all image preparation and measurements.

## Supporting information

S1 Table Dominant modifier RNAi screen for genetic interactions with FraFL. To find genes that potentially act downstream of Fra, UAS RNAi lines for each of the listed genes were crossed into the Ubx-GAL4, gal80ts, UAS-fraFL-myc background and adult eversion phenotypes scored.
(DOCX)
