# Supplementary material for: Frazzled can act through distinct molecular pathways in epithelial cells to regulate motility, apical constriction, and localisation of E-Cadherin
Source: PLoS One. 2018 Mar 8;13(3):e0194003. doi: 10.1371/journal.pone.0194003 (PMC5843272; doi:10.1371/journal.pone.0194003)
Supplement: S1 Table — To find genes that potentially act downstream of Fra, UAS RNAi lines for each of the listed genes were crossed into the Ubx-GAL4, gal80ts, UAS-fraFL-myc background and adult eversion phenotypes scored. (DOCX) [file pone.0194003.s001.docx]

# S1 Table. Dominant modifier RNAi screen for genetic interactions with FraFL.

| male genotype  (crossed to *UAS-fraFL-myc;Ubx-GAL4, gal80^ts^* females) | No defects (%) | Eversion  defects  (%) | Early  lethality  (%) | Total  (n) | p-value *  norm. vs  abnorm. | class |
| --- | --- | --- | --- | --- | --- | --- |
| *w1118* | 83 | 13 | 4 | 75 |  |  |
| *abl.IR (BL41710)* | 65 | 20 | 15 | 20 | 1.21E-01 | ns |
| *akt1.IR (BL33615)* | 0 | 0 | 100 | N/C | - | lethal |
| *aPKC.IR (BL34332)* | 0 | 0 | 100 | N/C | - | lethal |
| *baz.IR (BL35002)* | 45 | 10 | 45 | 31 | 2.46E-04 | enhance |
| *btk29a.IR (v25615)* | 49 | 8 | 43 | 49 | 1.20E-04 | enhance |
| *cadN.IR (BL41982)* | 0 | 0 | 100 | N/C | - | lethal |
| *cdc42.IR (BL42861)* | 75 | 25 | 0 | 12 | 6.88E-01 | ns |
| *cpa.IR (BL41685)* | 0 | 0 | 100 | N/C | - | lethal |
| *cpb.IR (BL41952)* | 61.3 | 6.4 | 32.3 | 31 | 2.45E-02 | enhance |
| *dab.IR (BL42646)* | 94 | 6 | 0 | 17 | 4.54E-01 | ns |
| *ds.IR (BL28008)* | 77 | 0 | 23 | 13 | 6.98E-01 | ns |
| *dscam.IR (BL38945)* | 13.5 | 13.5 | 73 | 37 | 1.26E-12 | enhance |
| *dscam2.IR (BL51839)* | 76.5 | 0 | 23.5 | 17 | 5.10E-01 | ns |
| *dscam4.IR (BL51508)* | 42 | 0 | 58 | 19 | 7.54E-04 | enhance |
| *ena.IR (BL39034)* | 0 | 0 | 100 | N/C | - | lethal |
| *ens.IR (BL40825)* | 46 | 18 | 36 | 28 | 4.56E-04 | enhance |
| *fak56d.IR (BL33617)* | 55 | 15 | 30 | 67 | 4.79E-04 | enhance |
| *fra.IR (BL31469)* | 97 | 0 | 3 | 60 | 1.20E-02 | suppress |
| *fps82d.IR (BL36053)* | 61.5 | 10 | 28.5 | 21 | 6.96E-02 | ns |
| *fps82d.IR (v107266)* | 55 | 21 | 24 | 38 | 3.06E-03 | enhance |
| *gef26.IR (BL28928)* | 29 | 33 | 38 | 24 | 2.31E-06 | enhance |
| *gef64c.IR (BL31130)* | 68 | 5 | 27 | 90 | 3.23E-02 | enhance |
| *IF.IR (BL38958)* | 0 | 0 | 100 | N/C | - | lethal |
| *lrrk.IR (BL32457)* | 55.5 | 11 | 33.5 | 18 | 2.48E-02 | enhance |
| *mew.IR (BL44553)* | 65 | 9 | 26 | 23 | 8.73E-02 | ns |
| *mys.IR* | 0 | 0 | 100 | N/C | - | lethal |
| *par6.IR (BL35000)* | 94 | 0 | 6 | 100 | 2.55E-02 | suppress |
| *pbl.IR (BL28343)* | 0 | 3 | 97 | 60 | 1.18E-25 | enhance |
| *pi3k.IR (v107390)* | 0 | 0 | 100 | N/C | - | lethal |
| *scar.IR (BL51803)* | 48 | 4 | 48 | 27 | 9.13E-04 | enhance |
| *shg.IR (BL32904)* | 41 | 27 | 32 | 34 | 2.93E-05 | enhance |
| *siz.IR (BL39060)* | 0 | 0 | 100 | N/C | - | lethal |
| *sli.IR (BL31467)* | 71 | 0 | 29 | 34 | 2.05E-01 | ns |
| *slik.IR (BL35719)* | 56 | 16 | 28 | 43 | 2.43E-03 | enhance |
| *slik.IR (BL43783)* | 0 | 0 | 100 | N/C | - | lethal |
| *slik.IR (BL43784)* | 56 | 8 | 36 | 59 | 1.03E-03 | enhance |
| *src42a.IR (v17643)* | 51.4 | 22.8 | 25.8 | 35 | 1.10E-03 | enhance |
| *src42a.IR (v100708)* | 0 | 0 | 100 | N/C | - | lethal |
| *src64b.IR (BL30517)* | 43 | 21 | 36 | 61 | 1.76E-06 | enhance |
| *stai.IR (BL53925)* | 0 | 0 | 100 | N/C | - | lethal |
| *stan.IR (BL35050)* | 38 | 0 | 62 | 45 | 9.42E-07 | enhance |
| *rab6.IR (BL35744)* | 72 | 20 | 8 | 25 | 2.60E-01 | ns |
| *rab11.IR (BL27730)* | 0 | 0 | 100 | N/C | - | lethal |
| *rac1.IR (BL34910)* | 47.5 | 12.5 | 40 | 40 | 1.91E-04 | enhance |
| *rho1.IR (BL32383)* | 0 | 0 | 100 | N/C | - | lethal |
| *rhogap68f.IR (BL41990)* | 31 | 18 | 50 | 44 | 3.78E-08 | enhance |
| *rhoGEF2.IR (BL34643)* | 12 | 47 | 41 | 34 | 1.37E-12 | enhance |
| *tao1.IR (BL34881)* | 0 | 0 | 100 | N/C | - | lethal |
| *trim9.IR (v21405)* | 0 | 0 | 100 | N/C | - | lethal |
| *trim9.IR (v21406)* | 0 | 0 | 100 | N/C | - | lethal |
| *trim9.IR (v100767)* | 53 | 27 | 20 | 15 | 3.56E-02 | enhance |
| *trio.IR (BL43549)* | 53 | 15.5 | 31.5 | 19 | 1.25E-02 | enhance |
| *tum.IR (BL35007)* | 52 | 0 | 48 | 65 | 1.29E-04 | enhance |
| *unc5.IR (BL33756)* | 57 | 0 | 43 | 14 | 6.82E-02 | ns |
| *βIntν.IR (BL28601)* | 46 | 16 | 38 | 85 | 1.46E-06 | enhance |

All crosses were performed at 29*◦*C. N/C indicates “not counted” and refers to larval/embryonic lethality cases. * Values represent Fisher’s exact test values of normal progeny *vs* abnormal (i.e eversion defects or early pupal lethality).
